# Tuning the Properties of MOF‐808 via Defect Engineering and Metal Nanoparticle Encapsulation

**DOI:** 10.1002/chem.202005050

**Published:** 2021-03-16

**Authors:** Rifan Hardian, Stefano Dissegna, Aladin Ullrich, Philip L. Llewellyn, Marie‐Vanessa Coulet, Roland A. Fischer

**Affiliations:** ^1^ CNRS MADIREL (UMR 7246) Aix-Marseille University Campus St Jérôme 13013 Marseille France; ^2^ Chair of Inorganic and Metal–Organic Chemistry Catalysis Research Center Dept. of Chemistry Technical University of Munich Ernst-Otto-Fischer-Straße 1 85748 Garching Germany; ^3^ Institute of Physics University of Augsburg Universitätsstrasse 1 86159 Augsburg Germany

**Keywords:** adsorption, defects, encapsulation, MOF-808, reactivity

## Abstract

Defect engineering and metal encapsulation are considered as valuable approaches to fine‐tune the reactivity of metal–organic frameworks. In this work, various MOF‐808 (Zr) samples are synthesized and characterized with the final aim to understand how defects and/or platinum nanoparticle encapsulation act on the intrinsic and reactive properties of these MOFs. The reactivity of the pristine, defective and Pt encapsulated MOF‐808 is quantified with water adsorption and CO_2_ adsorption calorimetry. The results reveal strong competitive effects between crystal morphology and missing linker defects which in turn affect the crystal morphology, porosity, stability, and reactivity. In spite of leading to a loss in porosity, the introduction of defects (missing linkers or Pt nanoparticles) is beneficial to the stability of the MOF‐808 towards water and could also be advantageously used to tune adsorption properties of this MOF family.

## Graphical abstract

**Defects and metal nanoparticle** encapsulations have been shown to have significant impacts on structure, texture, morphology and reactivity of the synthesized MOFs. Control over defects creation and metal nanoparticle encapsulations can become useful avenues to adjust the properties of MOFs.

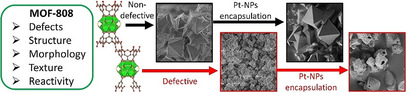

## Introduction

Metal–organic frameworks (MOFs) have attracted much attention in recent years due to their use in a wide range of applications from gas storage and separations,[^1^, ^2^] catalysis,[^3^, ^4^, ^5^] mechanics,[^6^, ^7^] to drug delivery,^[8]^ to name just a few. MOFs are a subclass of coordination polymers constructed from metal oxo‐clusters connected via organic linkers to form a 3D porous framework.^[9]^ The wide library of both organic and inorganic components leads to an almost infinite window to design MOF structures and chemistries.^[10]^ In particular, controlling the properties of MOFs is of significant importance and many strategies have been used in this regard. Some of these include the incorporation of mixed linkers,^[11]^ mixed metals^[12]^ and linker functionalization^[13]^ into the MOF structure. A more recent concept to improve MOF properties is to use defect engineering.[^14^, ^15^, ^16^] For instance, in Zr‐based MOFs, such as UiO‐66,[^17^, ^18^] the removal of a few linkers from the framework is not detrimental to its structural integrity,^[19]^ whilst it improves its reactivity and catalytic activity.[^16^, ^20^] The increased accessibility of metal sites as the linkers are removed has been proposed as the mechanism at the origin of these improvements.[^21^, ^22^] More recently, the tuning of the hydrophilicity of MOF crystals via defect engineering for efficient oil/water separation has been demonstrated in Zr‐based UiO‐66.^[23]^ The enhancement in catalytic activity has also been achieved by preparing a so‐called “composite MOF” or “metal@MOF” by metal impregnation^[24]^ or metal encapsulation.[^25^, ^26^] Similarly, tuning of the catalytic properties of MFI‐type zeolites has been suggested following the incorporation of Mo at defect sites within the structure.^[27]^ In this case, metal incorporation equally rendered the zeolite more hydrophobic. In the case of MOFs, metal encapsulation has been considered to be more advantageous than metal impregnation in retaining the encapsulated metal inside the MOF to prevent leaching during use.^[26]^ Nevertheless metal encapsulation may also be detrimental to the system since it may lead to metal deposition on the external surface of the MOF or to a framework degradation due to the formation of large nanoparticles.^[26]^

Apart from the extensively studied UiO‐66, there exist other zirconium‐based MOFs with a different number of connectivity's and variable topologies.[^17^, ^28^] One can cite NU‐1000 that has an 8‐connectivity with *csq* topology^[29]^ and MOF‐808 characterized by a 6‐connectivity with *spn* topology.^[30]^ MOF‐808 with hexanuclear zirconium is of particular interest because of its lower node‐connectivity that can provide more accessible open metal sites, especially if missing linker defects are engineered into the framework. This MOF is constructed from zirconium clusters linked together through trimesic acid or benzene tricarboxylic acid (BTC).^[31]^ Its micropore size distribution (pore size ≈1.8 nm) and open metal sites were shown to be useful for some catalytic applications, such as the hydrogenation of ethyl levulinate to γ‐valerolactone,^[18]^ the hydrolysis of nerve agent simulants,^[31]^ and in the Meervein–Poondorf–Verley (MPV) reaction.^[32]^

In this work defect engineering, metal encapsulation and both effects combined are investigated in the case of MOF‐808. The synthesised samples are first characterised in terms of structure, morphology, and texture. Water adsorption is used to follow variations in hydrophobicity of the samples and further changes in activity are probed via CO_2_ microcalorimetry that allows for a direct access to the adsorption enthalpy. This investigation highlights the complex interplay between the synthesis, morphology, texture, and chemistry of the materials which influence their sorption properties.

## Results and Discussion

### Synthesis

The general procedures to synthesize MOF‐808 samples were adapted from refs. [32] and [35]. Pristine MOF‐808 (noted MP) was synthesized using ZrOCl_2_.8H_2_O and H_3_BTC with molar ratio 1:1 and with a synthesis times of 7 days. The structural defects were introduced by modifying both reaction time and metal to linker ratio. The defective MOF‐808 (noted MD) was synthesized with a precursor molar ratio of 3:1 with a synthesis times of 2 days. All the details of the synthesis are given in the Supporting Information (section 1).

The effect of metal encapsulation was investigated using Pt nanoparticles (Pt‐NPs). The nanoparticles were synthetized using H_2_PtCl_6_.6H_2_O as Pt precursor and PVP as stabilizer The procedure was adapted from refs. [36, 37, 38], and is described in Supporting Information (section 2). Prior to their use in the MOF synthesis, Pt nanoparticles (Pt‐NPs) were characterized in depth (section 2 in Supporting Information), and TEM observations evidenced that the Pt nanoparticles possess an average diameter of 3.5 nm. The as synthetized Pt‐NPs were incorporated during the synthesis of MOF‐808 to produce Pt‐encapsulated pristine MOF‐808 (Pt@MP) and Pt‐encapsulated defective MOF‐808 (Pt@MD). All the details in terms of reactant quantities and synthesis procedure are given in Supporting Information (section 1 and Table S1). Table 1 summarizes the various synthetized samples together with some of their relevant characteristics for the present work.

**Table 1:** Presentation of the samples, summary of their synthesis conditions and number of missing linkers. Note that the number of missing linkers was calculated using TGA (see later) following the procedure detailed in Supporting Information (section 4).

Name	Description	Synthesis duration	Zr:BTC ratio	Missing linkers
MP	Pristine MOF‐808	7 days	1:1	≈1
MD	Defective MOF‐808	2 days	3:1	≈1.5
Pt@MP	Pt encapsulated in pristine MOF‐808	7 days	1:1	≈1
Pt@MD	Pt encapsulated in defective MOF‐ 808	2 days	3:1	≈2

### Phase purity and crystallinity

The removal of DMF is shown to be completed after activation under vacuum to 373 K. This is confirmed by Fourier‐transform infrared (FTIR) measurements (Figure S4 in Supporting Information). Indeed, the vibration band corresponding to the C−N vibration of DMF located at 1256 cm^−1^, is not observed in any of the synthesized samples. The X‐ray diffraction (XRD) patterns of all the samples (Figure S5 in Supporting Information) reveal that only the diffraction peaks corresponding to the cubic *Fd* $\bar{3}$ *m* structure of MOF‐808 are observed as confirmed by the simulated diffractogram.[^32^, ^33^, ^34^] It is worth noting that, in the case of Pt@MP and Pt@MD, no Bragg peaks of platinum are evidenced. This is linked to the nano‐size characteristics of the Pt‐NPs (Supporting Information, section 2). The instrumental broadening at high angles together with the quantity of Pt in the composite material make the detection of the Pt diffraction lines impossible. The presence of platinum is nevertheless confirmed by TEM observations and by ICP analysis that gave a quantity of Pt equal to 0.1 wt % and 0.04 wt % for Pt@MP and Pt@MD, respectively.

### Effect of synthesis conditions on crystal morphology

**Figure 1:**
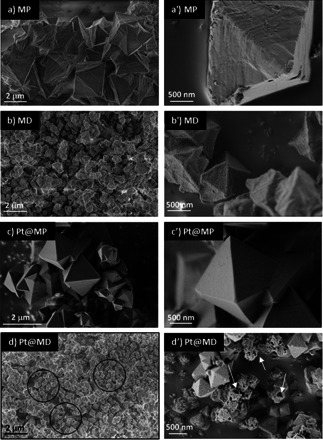
SEM images of the different synthetized samples: MP (a,a’); MD (b,b’); Pt@MP (c,c’); Pt@MD (d,d’). In Figure d) and d’), the circles and the arrows indicates the holes created in the MOF‐808 crystals.

Scanning electron microscopy (SEM) was used to gain insight into the crystal morphology. As seen in Figure 1, different synthesis conditions result in different crystal morphologies. Reactant stoichiometry strongly affects the crystal morphology. These effects can be observed by comparing the SEM images of MP (Figure 1 a and a’) with the ones of MD (Figure 1 b and b’).

Both samples show regular octahedral crystal shapes. In the case of MP, the crystal sizes are not uniform although they seem dominated by slightly larger crystals of about 2 μm. Sample MD has smaller crystal sizes with a more uniform size around 0.8 μm. This may be linked to the shorter reaction time in MD synthesis that is not enough for the growth process to produce the larger crystals. Indeed, longer reaction times would allow more nutrients in the solution to be incorporated into the growth phase as it has been observed in zeolite synthesis.^[39]^ Interestingly, MD shows less agglomerated crystals and this may be linked to the excessive amount of zirconium precursor in this particular synthesis reaction. Indeed, a proposed mechanism for MOF formation consists of an initiation stage through the deprotonation of organic linkers and dissociation of the metal salt followed by a complexation of the deprotonated organic linkers with the metal ions.^[40]^ Whereas the organic linkers are provided directly as a reactant, the metal precursors have to form the secondary building unit (SBU) in the synthesis process prior to combining with the organic linkers. The formation of the SBU is reported to be crucial for the MOF assembly process.^[41]^ Thus, the excessive amount of zirconium precursor could increase the probability of SBU formation that could in turn facilitate the formation of the MOF crystals.

### Effect of Pt‐NPs encapsulation on crystal morphology

By comparing the SEM images of samples MP (Figure 1 a and a’) and Pt@MP (Figure 1 c and c’), it can be observed that the presence of Pt‐NPs strongly influences the formation of MOF‐808 crystals: they possess a more regular shape and less roughness. However, the crystal size distribution remains heterogeneous in both cases. In terms of crystal size, Pt@MP has approximately 20 % smaller average size than MOF‐808 without platinum encapsulation MP. It can be proposed that platinum nanoparticles may act as seeds for MOF nucleation to occur. The zirconium ions may arrange around the platinum nanoparticle before coordinating with BTC and when the crystallization begins. This therefore leads to the platinum nanoparticles being encapsulated inside the MOF particles. As there are more nuclei, and sufficient linkers (1:1 ratio Zr:BTC), there will be more crystals but with smaller sizes. Similar mechanisms have been reported in the case of Keggin polyoxometalate (POM) encapsulated in several MOFs, such as Al‐MIL‐101‐NH_2_ and HKUST‐1.[^42^, ^43^] POM was also reported to assist the crystal formation of both Al‐MIL‐101‐NH_2_ and HKUST‐1, acting as nucleation sites and finally encapsulated inside the cage.

Other interesting features are observed when comparing sample MD (Figure 1 b and b’) with sample Pt@MD (Figure 1 d and d’). In this case, the combination of effects (excessive reactant stoichiometry and Pt‐encapsulation) eventually creates holes in most of the particles (see circles and arrow in Figure 1 d and d’) and generates a high degree of heterogeneity in crystal size. Those holes may be linked to (i) some Pt‐NPs that leave the crystal or (ii) to the free PVP introduced during the synthesis or (iii) to an incomplete coverage of the MOF around the Pt seeds.

### Pt‐NPs distribution in MOF‐808

**Figure 2:**
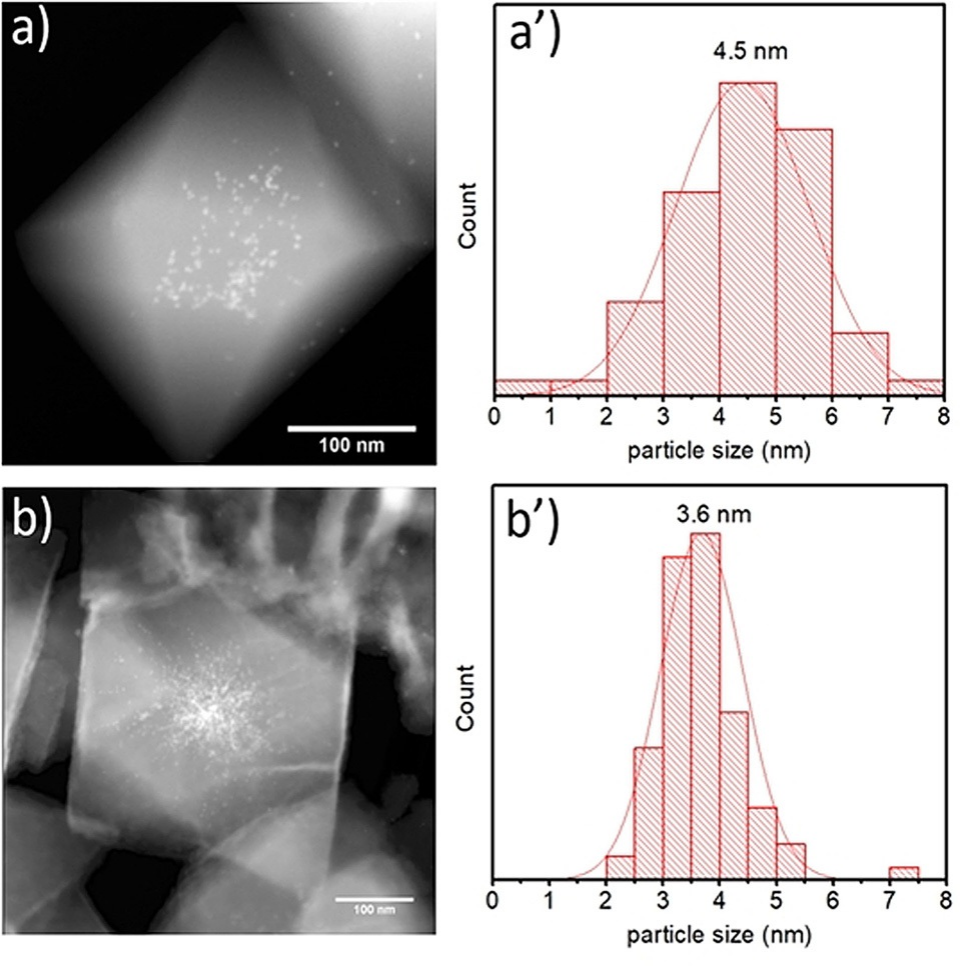
STEM images of Pt@MP (a) and Pt@MD (b) samples. The white dots located in the centre of the crystal indicate the presence of Pt‐NPs. Pt particle size distributions are given by the histograms for Pt@MP (a’) and for Pt@MD (b’). The distribution was made over 100 particles.

The presence of Pt‐NPs inside the MOF‐808 samples is evidenced using Scanning TEM (STEM) as shown in Figure 2. It can be observed that the particles are reasonably distributed inside the crystal and almost no agglomerates are visible. Since only a small contribution of Pt‐NPs could be observed bulging out from MOF edges, it suggests that most of the Pt‐NPs are encapsulated inside the MOF. The distribution of the Pt nanoparticles in Pt@MP is rather uniform and follows a normal distribution (Figure 2 a’) with an estimated mean size of around 4.5 nm. The fact that the mean size of the nanoparticles is slightly larger than their initial size prior to encapsulation indicates a slight nanoparticle growth during the formation of Pt@MOF‐808. This may be linked to a partial removal of the PVP during the MOF synthesis. It is worth noting that the size of the Pt‐NPs exceeds the characteristic pore size of the MOF‐808 (1.8 nm).^[33]^ This is not unexpected given the initial size of the Pt‐NPs. Such observations have already been made for both metal‐impregnated and metal‐encapsulated MOFs.^[25]^ One can expect that this observation may result in local defects or deformation of the host framework.

Nevertheless, the overall structural integrity of the MOF is not affected as can be observed from the sharp Bragg diffraction peaks (Figure S5 in Supporting Information). In the Pt@MD sample, the Pt‐NPs are also encapsulated inside the crystal but they seem more concentrated in the centre of the crystal (Figure 2 b). The average diameter of the Pt‐NPs was estimated in the same manner as for the Pt@MP sample and the particle distribution is given in Figure 2 b’. One can observe that their mean value is slightly smaller than in Pt@MP. One possible explanation is that the PVP capping agent may be removed due to longer reaction time for Pt@MP. In contrast, some PVP is still remaining in the Pt@MD preventing the formation of larger Pt‐NPs. Unfortunately, due to the encapsulation of Pt(PVP)‐NPs inside the MOF crystal, the peaks of PVP in the Pt@MOFs samples are not easily distinguishable in FTIR analysis (Figure S4 in Supporting Information).

### Thermal and structural stability

**Figure 3:**
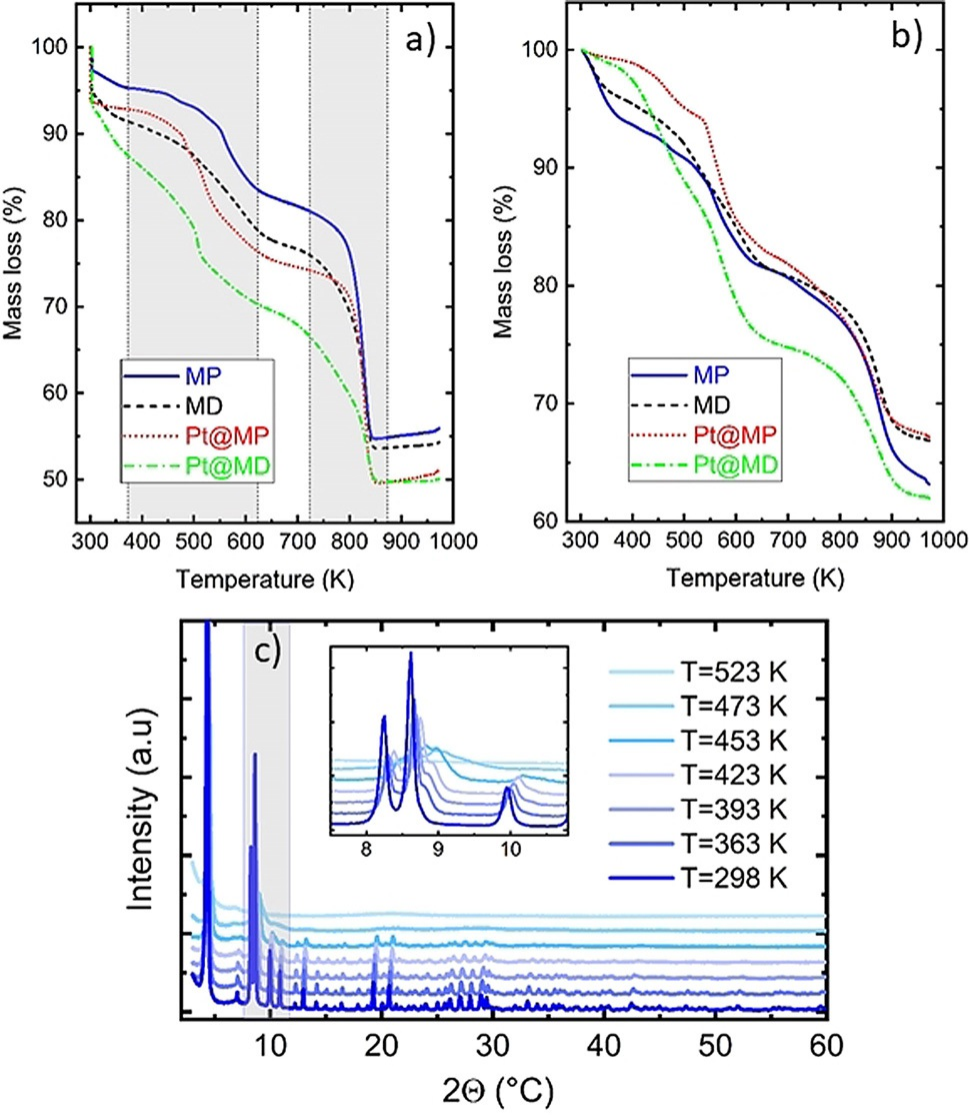
TGA of the activated samples MP, MD, Pt@MP and Pt@MD performed under air flow (a) and under nitrogen (b) at a heating rate of 5 K min^−1^. The dotted lines drawn in (a) are guidelines to indicate the various steps in decomposition. (c) X‐ray diffraction patterns acquired at 1.54 Å for MP sample. The heating was carried out under nitrogen from room temperature to 523 K.

The stability of the activated materials was followed using thermogravimetry analysis (TGA) and variable temperature XRD (VTXRD). The combination of these methods allows for a clearer understanding of the correlation between mass loss (species removal) and structure integrity. TGA analyses were carried out under synthetic air (Figure 3 a) and under nitrogen flow (Figure 3 b). The analysis of mass decomposition was performed using the experiments carried out under air flow since it guarantees a complete oxidation of the samples. As can be seen in Figure 3 a and 3b, below 373 K, a visible mass loss is observed for almost all samples (between 2 and 5 %). It can be attributed to the removal of water or other loosely bound species adsorbed on the external surface of the MOFs. For the experiments performed under Air, in the case of Pt@MD, a larger mass loss is observed (around 8 % at 373 K) and may be correlated to the larger external surface area (see Table 2 in next section). This is consistent with the SEM analysis showing the presence of holes in several Pt@MD crystals (Figure 1 d and 1d’).

**Table 2:** Summary of the physisorption data obtained using nitrogen adsorption measurements for the different samples. The BET method was applied in *p*/*p* ^0^ range between 0.05 and 0.1. The pore volume is given at *p*/*p* ^0^=0.7 for all of the samples. The external area were calculated using the t‐method.

Name	BET area [m^2^ g^−1^]	Pore volume [cm^3^ g^−1^]	External area [m^2^ g^−1^]
MP	1680	0.69	10
MD	1597	0.67	28
Pt@MP	1538	0.61	15
Pt@MD	933	0.41	64

It is noticeable that, while there is a small plateau around 373 K for the non‐defective samples, it is lacking in the case for MD and Pt‐MD for which the mass continuously decreases with increasing the temperature. In the temperature region between 373 K and 623 K, the mass loss can be attributed to the removal of coordinated ‐OH and H_2_O that replace the formic acid after activation.^[31]^ Indeed, the formic acid was used during the synthesis as modulator and was removed during activation. Nevertheless, we cannot exclude the possibility of incomplete formic acid replacement. Other work attributed these features to the loss of any remaining DMF solvent that has not been completely exchanged and/or evaporated.^[28]^ However, in the present case FTIR analysis strongly suggests that DMF has been completely removed from the samples (Figure S4 in Supporting Information). The last notable region in the TGA curve is located between 723 K and 873 K. It corresponds to the BTC linker decomposition. Quantifying the mass loss in this region can give information on the quantity of missing linkers in each sample. The methodology to estimate the missing linker concentration was adapted from ref. [28] and is explained in detail in Supporting Information. Assuming a similar mechanism to UiO‐66, at high temperature (about 673 K), the coordinated water and hydroxyls, that replace the formate, are removed. According to Moon et al.,^[31]^ this would lead to the activated MOF‐808 with a formula of Zr_6_O_8_[(C_6_H_3_)(COO)_3_]_2_ and a molar mass equal to 1095.6 g mol^−1^. Assuming that the final residue is (ZrO_2_)_6_ (Mm=739.32 g mol^−1^),^[28]^ the theoretical linker loss can be estimated. The obtained results are given in Table 1. One can observe that for all samples, the mass losses are lower than the theoretical ones for a perfect MOF‐808. This suggests that even the pristine materials MP may already contain inherent missing linker defects. It is noticeable that excessive zirconium ratio (MD and Pt@MD) results in a higher concentration of missing linker defects. Although the mechanism of this effect on the formation of missing linkers is still unclear, as discussed above, it is possible to propose that the rate of SBU formation is accelerated by the excessive amount of zirconium precursor and increase the competition to assemble with a more limited number of organic linkers. This in turn may result in an incomplete MOF structure formation containing missing linker defects.

VTXRD experiments were used to follow the structural changes upon heating and the consequences of the removal of chemical species on the structural integrity. An example of one VTXRD pattern is presented in Figure 3 c for sample MP. It is noticeable that the sharp Bragg peaks are maintained up to around 423 K, temperature at which the loss in crystallinity starts as evidenced by the enlargement of the diffraction lines. At 523 K, the increase of the scattered intensity at low 2*θ* values may be the signature of the sample amorphization. In the literature, the temperature at which the structural degradation occurs is reported at different values. Moon et al.,^[31]^ reported the structural collapse of MOF‐808 above 523 K under air, while Plessers et al.,^[32]^ reported structural retention up to 423 K under vacuum with degradation occurring between 423 K and 473 K. These latter values are consistent with the ones observed in this study. Moreover, this temperature range is far below the temperature of the BTC linker removal, which occurs at around 773–873 K. This suggests that the removal of chemical species below 673 K may be responsible for the structural degradation. By comparing the TGA results (Figure 3 b) with VTXRD patterns (Figure 3 c), it can be proposed that structural degradation occurs in the temperature region where either coordinated H_2_O and ‐OH or formic acid are removed. This suggests that the removal of these coordinated substances could be responsible for the loss of long‐range order. Although the connectivity between the SBU to form a framework is maintained by BTC linkers and neither by formic acid nor H_2_O and OH, the removal of those latter species may, nevertheless, destabilize the structure.

### Textural properties

**Figure 4:**
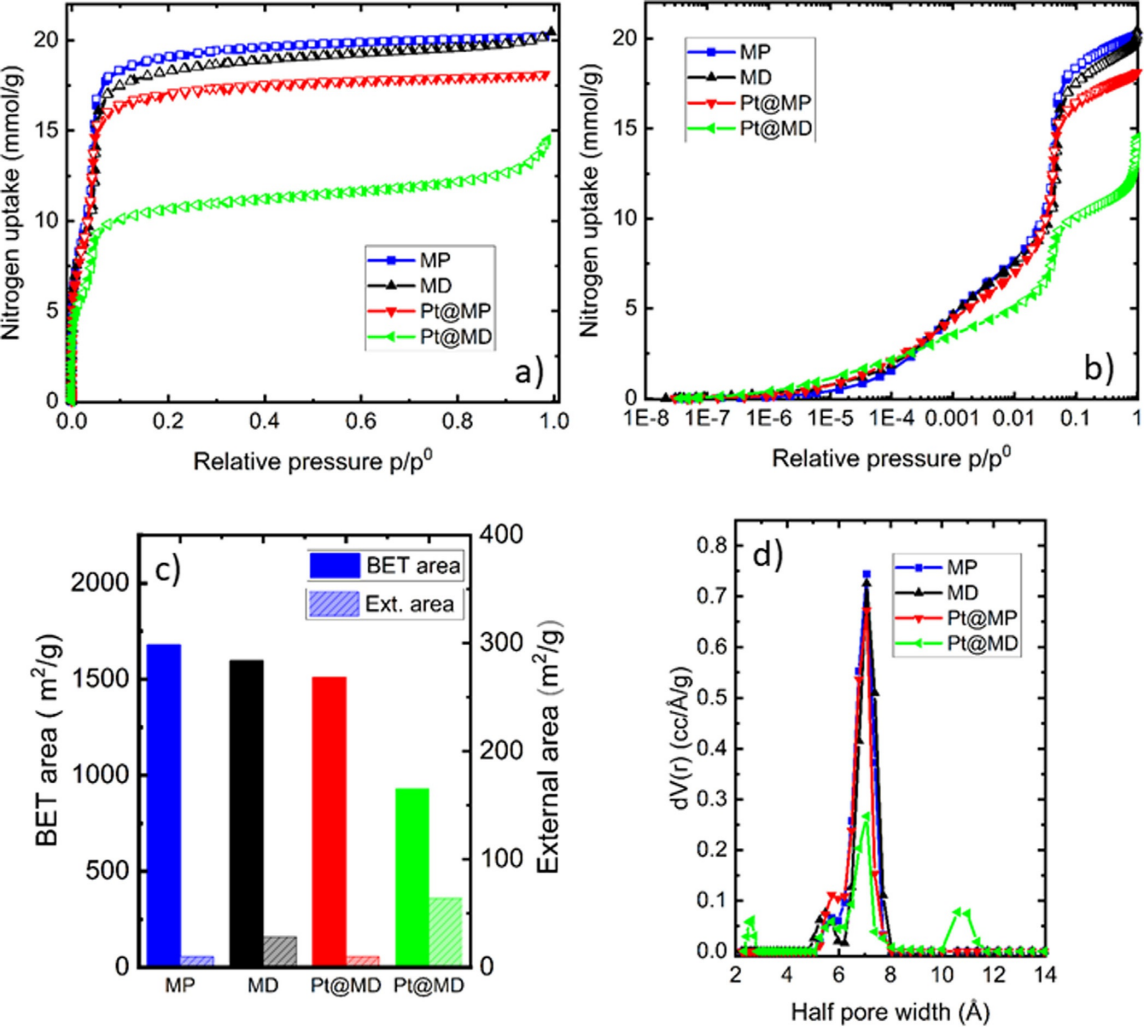
Results obtained from N_2_ sorption measurement performed at 77 K. (a, b) N_2_ adsorption–desorption isotherms, (c) BET area and external area, (d) pore size distributions calculated by QSDFT using kernels of nitrogen adsorption on carbon slit and cylindrical pores.

The texture of the synthesized materials was characterized by means of nitrogen physisorption at 77 K in order to evaluate the surface area and porosity. The adsorption‐desorption isotherms are presented in Figure 4 a and b. According to the last IUPAC classification,^[44]^ they are between type I(b) and type IV(b) in shape which suggests they are characteristic of microporous systems having a narrow pore size distribution that are filled and unfilled at the same relative pressure. These observations are consistent with literature on MOF‐808.[^33^, ^34^, ^45^] The defective and/or impregnated samples present an additional uptake in the very last part of the isotherm (*p*/*p* ^0^>0.9) and this step is more obvious in the case of Pt@MD. It may be related to the roughness of the crystal surface, the presence of holes (Figure 1 d and 1d’) and to the particle sizes that are all responsible for an increase in the external surface area. Such behaviour was reported in defective and impregnated MOF 808 but also in pristine MOF 808.[^45^, ^46^, ^47^, ^48^] The surface area (BET method) and the pore volume at *p*/*p* ^0^=0.7 were calculated for each sample and the values are tabulated in Table 2. The external surface area calculated using the *t*‐method is also given in Table 2 and represented in Figure 4 c together with the BET areas. The pristine material MP shows the highest BET area. The values obtained in this study are consistent with ones published in the literature which vary between 1205 m^2^ g^−1^ and 2060  m^2^ g^−1^ for pristine MOF‐808 samples.[^33^, ^34^, ^46^, ^48^, ^49^]

The effects of different reactant stoichiometries can be studied by comparing MP and MD. Both BET areas and pore volume are slightly lower for the defective sample MD. This is a surprising result since it has been observed in other MOFs systems such as UiO‐66,^[16]^ that the presence of missing linkers tends to increase the surface area and the pore volume. However, in the present case the shorter reaction time in the case of MD may have led to an incomplete formation of some of the crystals.

Pt‐encapsulation inside the pristine sample MP also induces a slight reduction in BET area. It can be correlated to the fact that metal encapsulation may block some of the pores.^[25]^ Such an effect has been observed for Pd‐modified MOF‐808^[47]^ and imidazole‐MOF‐808.^[45]^ Nevertheless, the small reduction in surface area could equally be linked to the difference in densities and in the quantity of Pt that contributes to the nitrogen sorption. Finally, the case of Pt@MD is typical as it highlights a combination of effects. The textural properties (BET area and pore volume) of this sample are the lowest. This could be caused by two factors: (i) Pt‐nanoparticles that occupy the pores as in the case of Pt@MP and (ii) incomplete formation of the crystal as hypothesized above (Figure 1 d and d’).

The pore size distributions were determined using QSDFT kernels of nitrogen adsorption on carbon slit and cylindrical pores. The half pore width distribution is given in Figure 4 d. There are no major differences between the samples, that all possess a mean half pore size around 7 Å. This value corresponds well with the mean aperture diameter size of 14 Å given in in the literature.[^33^, ^34^]

### Activity assessment

**Figure 5:**
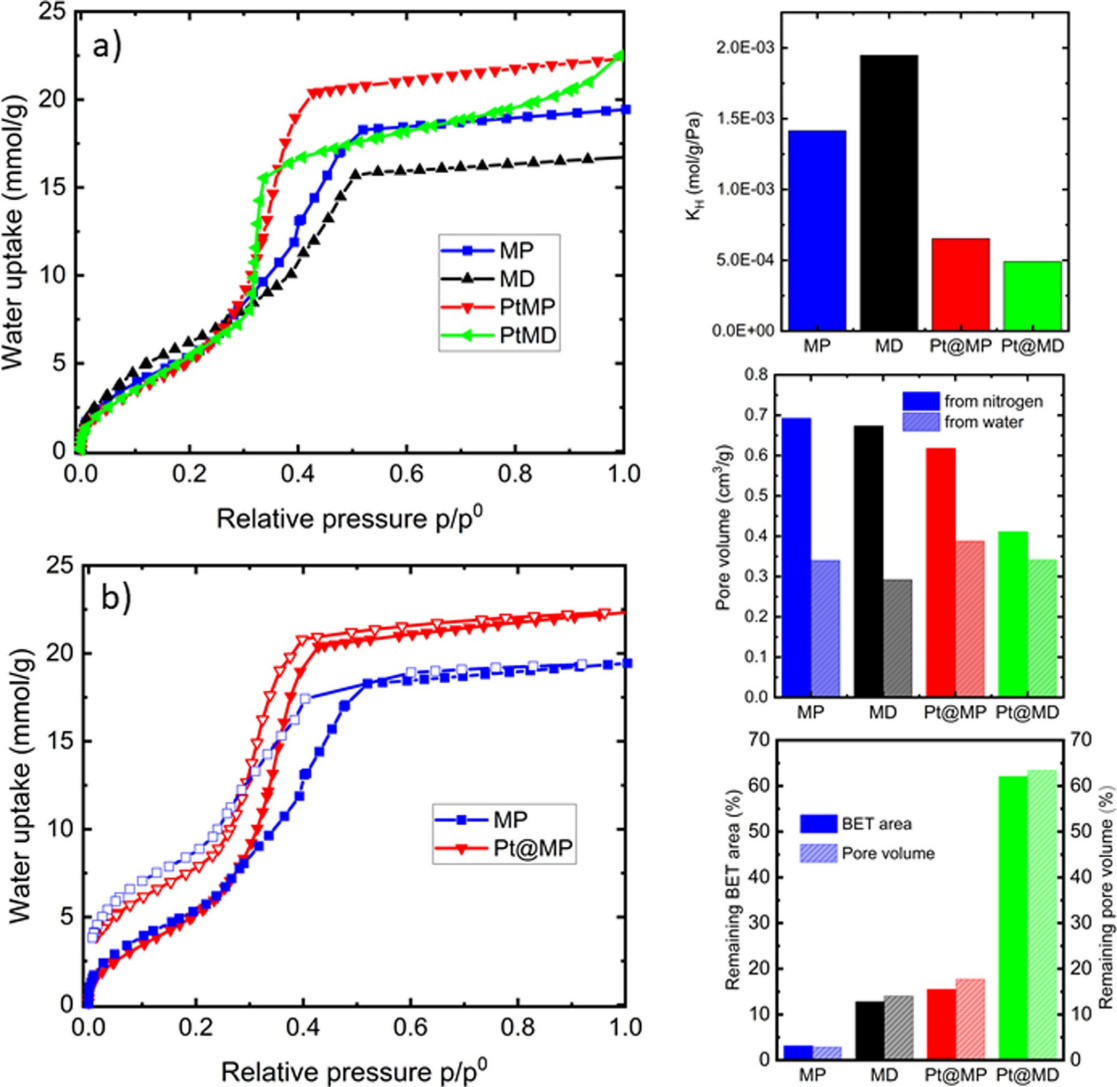
Results obtained from water sorption measurements performed at 298 K. (a) Water adsorption isotherms for all the samples. (b) Water adsorption‐desorption isotherms for pristine material MP and Pt@MP (c) Henry's constant K_H_ determined from the water adsorption isotherms in the low‐pressure regime. (d) Pore volumes obtained from N_2_ and water adsorption isotherms. (e) Remaining BET area and pore volume, obtained for N_2_ adsorption isotherm after one adsorption cycle of water.

Water adsorption has been proposed as a useful method to explore the chemistry of defective MOFs.^[16]^ Moreover it offers insight into the stability of MOFs toward water which is of utmost importance for being industrial applications of MOFs.^[50]^ The water adsorption isotherms of the various samples are presented in Figure 5 a and b. The complete set of adsorption‐desorption isotherms are given for all the samples in Figure S7 in Supporting Information.

At low relative pressure, where the adsorption occurs at the most energetic sites, the strength of the interaction can be estimated from the Henry's constant and this can equally be an estimation of the potential reactivity. The Henry's constants were calculated following previous works^[16]^ and the obtained values are summarized in Figure 5 c. It can be observed that the highest Henry's constant is obtained for MD sample and this can be attributed to the existence of missing linker defects, providing more accessible open metal sites which is consistent with previous works.^[16]^ The presence of Pt‐nanoparticles strongly reduces the activity of the samples and the combined effect of encapsulation and defect (Pt@MD) leads to the sample having the lowest interaction with water. This may suggest that the metal encapsulation blocks the access to the defect sites thus rendering the sample more hydrophobic with respect to the samples without Pt. This trend is similar to that observed during metal encapsulation in zeolites, as mentioned above.^[27]^

The first plateau region in the water adsorption isotherm (*p*/*p* ^0^ between 0.02 and 0.2 in Figure 5 a and 5b) corresponds to the completion of surface coverage. Pore filling occurs for *p*/*p* ^0^ values around 0.2 to 0.6; when significant variations in uptakes are evidenced. Interestingly, the slope of the pore filling step is steeper for the two samples with encapsulated than for the samples without platinum that evidence more gradual slopes. A explanation for such a behaviour is probably a more rapid water cluster formation for samples containing Pt.^[51]^ Furthermore, Figure 5 b shows a pronounced hysteresis in the water isotherms. It is known that water can lead to instability in many MOFs and even for so‐called stable MOFs, water can lead to pronounced reversible structural changes.^[52]^ Thus the observed hysteresis can be explained by a rehydroxylation of the sample or by reversible structural modifications during water adsorption.

One can compare the pore volume measured with nitrogen at 77 K and water vapor at 298 K. To convert the amount adsorbed at the plateau (*p*/*p* ^0^=0.6–0.8) into a pore volume, it is commonly assumed that the pores are filled with the condensed adsorptive in the bulk liquid state (Gurvich rule).^[53]^ The pore volumes calculated at *p*/*p* ^0^=0.7 are given in Figure 5 d and it can be observed that volumes obtained with water are systematically lower than those obtained with nitrogen and the difference is smaller for Pt‐containing samples. This tendency could be explained by (i) the specific mechanisms associated to water adsorption such as clustering or even structural contraction,^[52]^ (ii) the presence of hydrophobic domains within the pores or (iii) the deterioration of the MOF structure with water. Indeed, water adsorption in MOF‐808 has been reported to strongly deteriorate their structure.^[33]^ In order to quantify this effect in the samples of this study, nitrogen sorption isotherm was performed on the same batch after the water sorption cycle. The remaining BET area and pore volume calculated from those isotherms can be used to monitor the extent of the deterioration of the samples. The obtained values are presented in Figure 5 e. It is noticeable that both the BET area and pore volume follow the same trends which means that the undamaged part of the sample keeps its porosity. Moreover, one can observe that, while the pristine material is strongly damaged after water adsorption, the deterioration is lower for the modified samples; the less damaged sample being the defective and Pt encapsulated sample Pt@MD.

### Activity assessment—CO_2_ adsorption microcalorimetry at 303 K

**Figure 6:**
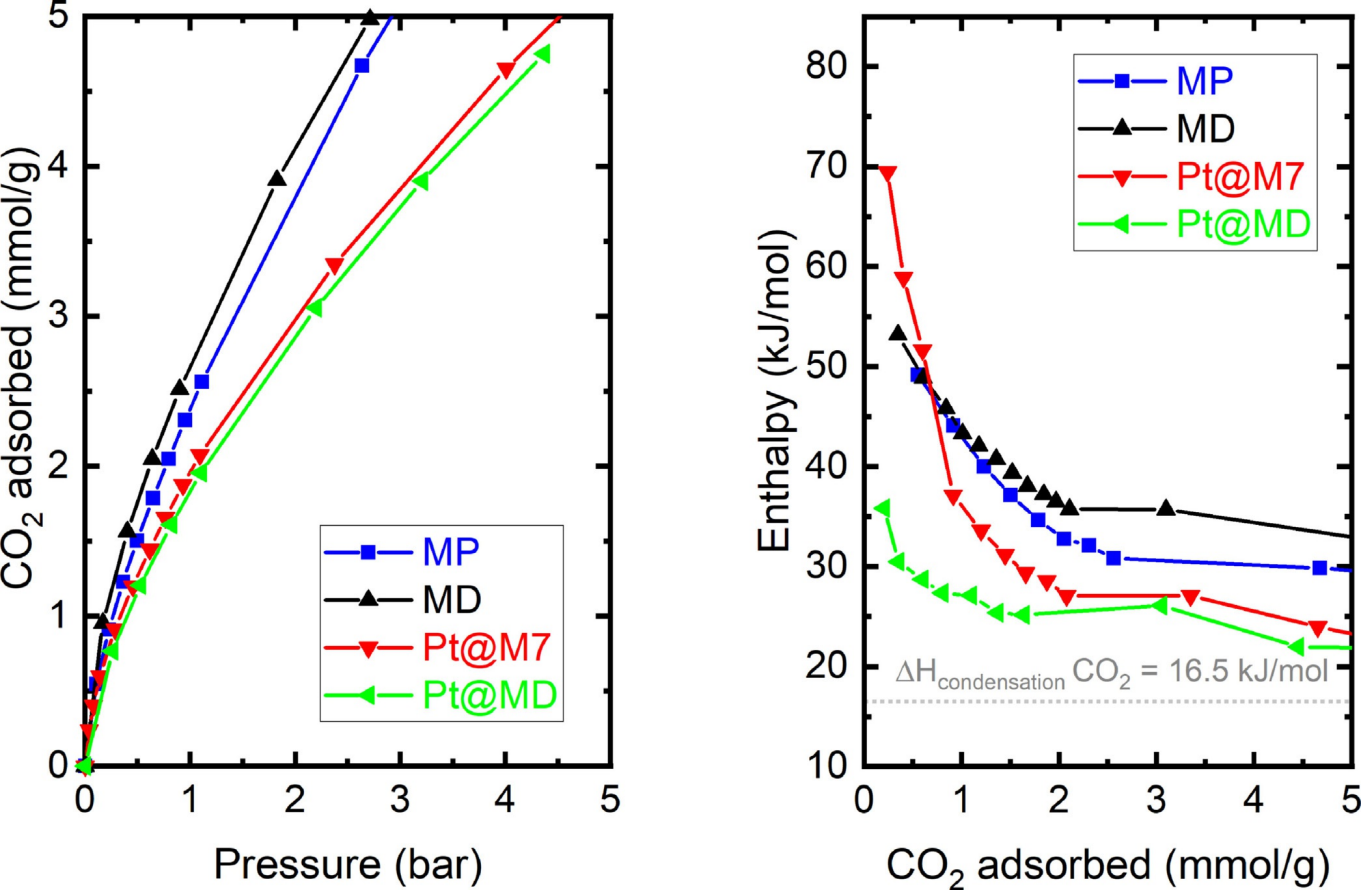
CO_2_ adsorption isotherms at 303 K (a) and adsorption enthalpies (b).

CO_2_ is an ideal probe, due to its quadrupole moment, to explore the surface activity of porous materials. The CO_2_ adsorption isotherms at 303 K for all studied samples and their corresponding adsorption enthalpies are presented in Figure 6.

The interactions between adsorbate–adsorbent can be well monitored at very low coverage and give indications concerning the adsorbent affinity towards this probe.^[54]^ In this region, the interaction of an adsorbate molecule with an energetically homogenous surface will give rise to a constant calorimetric signal. On the other hand, a decreasing signal will be observed in the case of interactions between an adsorbate molecule with an energetically “heterogenous” surface.[^53^, ^55^]

The energetically heterogenous surface may arise from the pore size distribution and/or varying surface chemistries.^[56]^

In the case of the samples discussed in this work, all of the CO_2_ enthalpies show decreasing enthalpies up to a coverage of around 2 mmol g^−1^, suggesting that specific sorption sites are available for the CO_2_ probe. The CO_2_ adsorption isotherms (Figure 6 a) shows two groups for the samples with or without the Pt nanoparticles. The MOF‐808 samples without the Pt‐NPs show higher uptakes than those loaded with the Pt‐NPs. Again, this could be linked to pore blocking or to the difference in the sample densities. Unlike in nitrogen adsorption at 77 K and water adsorption at 298 K, stepped isotherms are not observed for the measurement of CO_2_ adsorption at 303 K. This is correlated with the different adsorption mechanisms in sub‐critical states (nitrogen at 77 K and water at 298 K) and close to the super‐critical state (CO_2_ at 303 K). In the former state, the adsorbed liquid can reach relative pressures where pore filling can occur, whereas for CO_2_, adsorption is essentially restricted to monolayer edification.

Comparing the samples without the Pt‐NPs, the defective sample MD shows lightly higher uptakes with respect to the MP sample. This slight difference between the samples is less visible in the calorimetry data. Indeed, the adsorption energies at zero coverage obtained by linear interpolation lead to adsorption energies equal to 57 kJ mol^−1^ for MD and 56 kJ mol^−1^ for MP. This difference could be related to the number of defects, presumably missing linkers as discussed above. However, one must underline that those energy differences are quite moderate with respect to the energy differences for samples containing the Pt‐nanoparticles. The CO_2_ isotherms obtained with the Pt‐containing samples are quite similar. This could be the result of adsorption at similar sites. However, the initial adsorption energies are significantly different. With sample Pt@MP, the initial adsorption enthalpy is the highest observed in this sample set. This highlights the potential role of the Pt nanoparticles that strongly interact with the CO_2_. However, the calorimetric signals rapidly drop indicating that the number of active sites that govern this strong interaction is limited.^[57]^ Surprisingly, Pt@MD has the lowest initial adsorption enthalpy. This lowest energy is in accordance with the results of the Henry's constant obtained from water adsorption. Indeed, one would expect that the Pt would provide strong adsorption sites. One hypothesis, discussed above for water, may be that the metal nanoparticles are situated at the MOF defect sites. A second explanation is that the polymer protecting agent (PVP) initially present on the surface of the Pt‐nanoparticles is not completely removed during the synthesis of the Pt@MD and can, thus, somehow cover the active sites in this material (explaining the lower enthalpy), and some of the porosity (explaining the lower uptakes in the water and N_2_ isotherms).

## Conclusions

In this study, the synthesis and characterization of defective and Pt‐encapsulated MOF‐808 was presented. Structural defects such as missing linkers were introduced by using an excess of metal precursors in the reaction mixture. Pt‐nanoparticles have been successfully encapsulated in both pristine and defective MOFs. It is found that synthesis conditions (reaction time, reactant stoichiometry and encapsulating Pt‐nanoparticles) influence morphology, textural properties and affinity towards water and CO_2_.

The BET area of pristine MOF‐808 (MP) is higher than defective and Pt‐encapsulated MOF‐808 (Pt@MP). This indicates the roles of morphology and pore blocking on the textural properties of MOF‐808. Interestingly, Pt‐encapsulation on the pristine MOF‐808 seems to enhance the crystallinity of MOF‐808 since well‐defined octahedral crystal with smooth surface topography are observed.

Water adsorption, used to explore the hydrophilicity of the samples, demonstrate that the highest Henry's constant is obtained for defective MOF‐808 sample (MD). The presence of missing linker defects that provide more accessible open metal sites, are proposed to be responsible for this effect. Interestingly, the MOF‐808 stability towards water is systematically enhanced by the presence of missing linkers and/or Pt encapsulation. The sample that combines both effects (missing linkers and Pt encapsulation in Pt@MD) presents a remaining porosity after water adsorption close to 60 % (as compared with a few % for pristine sample.).

Probing the samples with CO_2_ shows slight differences upon defect introduction and/or Pt encapsulation. In general, the increased number of defects leads to slightly higher CO_2_ adsorption energies. Addition of Pt into the sample with fewer defects (Pt@MP) leads to increased interactions with CO_2_. The inverse observation is made for the sample containing the most defects (Pt@MD) which may suggest that the metal shields (or even anneals) the defect sites in the MOF.

To summarize, defect engineering of metal–organic frameworks can be achieved in the raw MOF‐808 material which leads to expected correlations of energy vs. number of defects. The introduction of metal nanoparticles leads to a decorrelation which could be the result of metal shielding the MOF defect sites.

## Experimental Section

**Characterization techniques**: Prior to their characterization, the samples were activated at 100 °C under vacuum. The structure of both platinum nanoparticles and MOF‐808 series were investigated using XRD. The measurements were carried out using a Panalytical Empyrean instrument working with a copper source (*λ*=1.54 Å). The patterns were recorded from 2*θ* between 3° and 50°. The same apparatus was used to perform XRD measurements upon heating in order to follow the thermal stability of the samples. The heating was carried out under a nitrogen atmosphere using an Anton Paar XRK 900 furnace. Combined with TGA analyses, XRD measurements allowed some insight to be obtained on the chemical species that are responsible for the structural degradation of MOF‐808.

Fourier‐transform infrared (FTIR) was carried out using an ATR FTIR (PerkinElmer UATR Two). Data were collected from wavenumber 450 to 4000 cm^−1^. The samples were placed in a holder and measured without mixing with KBr.

The morphology of the MOF‐808 series was studied using Scanning Electron Microscopy (SEM). The SEM measurements were performed with a Zeiss Gemini 500 instrument from CP2M platform (FR 1339‐ CNRS‐AMU). The voltage was set between 0.5 to 5 kV to have optimum conditions leading to a good compromise between the suppression of the charge effect and lateral resolution. The working distance between camera and sample was set at 1.8 mm or 0.9 mm to enhance the resolution

Scanning Transmission Electron Microscopy (STEM) and high resolution TEM were used to characterize the Pt‐encapsulated samples. The measurements were performed using a Jeol 2100F working at 200 kV and a Gatan Digital Micrograph with a Digiscan II system. The grids for STEM measurement were prepared by dispersing a small amount of sample in methanol followed by sonification and decantation. Afterwards, a few drops were deposited on the dedicated copper grid and left in oven at 363 K for 1 hour to evaporate the methanol solvent.

Platinum loading in impregnated samples was measured by using Induced Coupled Plasma (ICP) analysis with a 7900 ICP‐MS (Agilent) equipped with a SPS4 autosampler (Agilent).

Thermal stability of the synthesized materials was studied by using thermogravimetric analysis (TGA). The analyses (TGA) were performed with a TA Q500 instrument using platinum crucibles under synthetic air flow of 100 mL min^−1^ and another set of experiments performed under nitrogen flow of 100 mL min^−1^. The analyses were initiated by applying an isotherm at room temperature (298 K) for 15 minutes followed by heating at a rate of 5 K min^−1^ up to 973 K.

Texture was analysed using nitrogen sorption at 77 K. The measurements were done using a BELSORP Max 1. The available surface area, pore volume, external surface and pore size distribution were deduced from the measurements. Prior to the measurements, the samples were activated to 373 K under vacuum for 17 hours.

Water vapor adsorption at 298 K was performed using a BELSORP Max 1. Adsorption equilibrium was assumed when the variation of the cell pressure was 0.5 % for a minimum period of 300 seconds. Prior to the measurements, the samples were activated to 373 K under vacuum for 17 hours.

CO_2_ adsorption microcalorimetry was carried out at 303 k using a lab‐built instrument.^[56]^ A Tian–Calvet type microcalorimeter, equipped with thermopile with around 900 chromel–alumel thermocouples, was used to directly measure the adsorption energies. Manometry is used to measure the adsorption isotherm using a stepwise introduction of the carbon dioxide. In the initial regions of loading, errors in the enthalpies are of the order of ±1 kJ mol^−1^. In these experiments, a thermal equilibration time was set at 200 minutes between each gas dose. Prior to the measurements, the samples were activated to 373 K under vacuum for 16 hours.

## Conflict of interest

The authors declare no conflict of interest.

## Supporting information

As a service to our authors and readers, this journal provides supporting information supplied by the authors. Such materials are peer reviewed and may be re‐organized for online delivery, but are not copy‐edited or typeset. Technical support issues arising from supporting information (other than missing files) should be addressed to the authors.

Supplementary
