# Supplementary material for: Tuning the Properties of MOF‐808 via Defect Engineering and Metal Nanoparticle Encapsulation
Source: Chemistry. 2021 Mar 16;27(22):6804–14. doi: 10.1002/chem.202005050 (PMC8251568; doi:10.1002/chem.202005050)
Supplement: Supplementary file 1 — Supplementary [file CHEM-27-6804-s001.pdf]

Chemistry–A European Journal

Supporting Information

Tuning the Properties of MOF-808 via Defect Engineering and Metal Nanoparticle Encapsulation

Rifan Hardian,[a] Stefano Dissegna,[b] Aladin Ullrich,[c] Philip L. Llewellyn,[a]
Marie-Vanessa Coulet,*[a] and Roland A. Fischer*[b]

1. Details of synthesis procedures

Materials used to the samples are $ZrOCl_2.8H_2O$ (Alfa Aesar, 98%), H_3BTC (ABCR, 98%), N,N-Dimethylmethanamide (DMF, Sigma-Aldrich, 99.8%), formic acid (FA, Sigma-Aldrich, 97%) and Methanol (Sigla-Aldrich, 99%).

The synthesis procedure of the pristine MOF-808 (**MP**) was adapted from the literature[1,2]. 960 mg $ZrOCl_2.8H_2O$ and 660 mg H_3BTC were each dissolved in 60 mL DMF, prior to mixing. In the next step, 120 mL of FA was added into the solution. All the solutions were mixed and put in an oven at 100°C for seven days to produce MP. The precipitated white powder and the remaining solution was removed by centrifugation. The product was washed with 25 mL DMF and centrifuged again. DMF washing was performed 3-4 times per day for 2 days. The washing procedure was continued by replacing DMF with methanol for 4 times per day for 2 days. The washed powders were then dried under atmospheric conditions and then activated at 100°C under vacuum overnight.

For the synthesis of defective MOF-808 (**MD**), the stoichiometric molar ratio between zirconium salt and BTC was made to be 3:1 following the work by Jiang,[2] Healey,[3] and Plesseres.[4] 970 mg of $ZrOCl_2.8H_2O$ and 210 mg of H_3BTC were respectively diluted in 20 mL and 25mL of DMF. After that, 45 mL of FA was added into the solution. All the solutions were mixed and put in an oven at 130°C for two days. The rest of the procedure was the same than for the previous sample.

The detailed procedure for the Pt nanoparticles' synthesis is given in section 2. Pt nanoparticles encapsulation procedure was the following. For the pristine MOF-808 containing Pt (**Pt@MP**), 60 mg of Pt NPs were diluted in 8 mL DMF and were added to 960mg of $ZrOCl_2.8H_2O$ dissolved in 52ml DMF. This solution was then mix with the solution containing 660 mg H_3BTC dissolved in 60 mL DMF. Then the same steps that for MP sample were followed.

For the defective MOF-808 containing Pt (**Pt@MD**), 60 mg of Pt NPs were diluted in 5 mL DMF and were added to 970mg of $ZrOCl_2.8H_2O$ dissolved in 20 ml DMF. This solution was then mix with the solution containing 210 mg H_3BTC dissolved in 20 mL DMF. Then the same steps that for MD sample were followed.

The experimental conditions for each synthesis are summarized in Table S1.

Table S1. Experimental condition used of the synthesis of the different products

Samples	$ZrOCl_2$ (mg)	H_3BTC (mg)	Zr:BTC ratio	CH_2O_2 (ml)	DMF (ml)	Pt-NPs (mg)	time (days)

**MP**	960	660	1:1	120	120	-	7
**MD**	970	210	3:1	45	45	-	2
**Pt@MP**	960	660	1:1	120	120	60	7
**Pt@MD**	970	210	3:1	45	45	60	2

2. Synthesis and characterization of Pt-nanoparticles (Pt-NPs)

The procedure to synthetize Pt nanoparticles was adapted from literature.[5–7] The reactants are PVP [6] (poly(vinyl pyrrolidine)), methanol, $H_2PtCl_6.6H_2O$, distilled water, acetone, diethyl ether, and ethanol. PVP is used because it is known to be an excellent stabilizer often used for the synthesis of nanoparticles.[8] Methanol plays role as reductant (reducing agent) in the presence of PVP that also acts as the capping agent to cover the Pt nanoparticle.[5]

186 mg of $H_2PtCl_6.6H_2O$ was used as a Pt precursor and diluted with 60 mL distilled water. 399 mg of PVP was dissolved in 540 mL of methanol. The Pt solution was poured into the PVP solution and placed under reflux at 80°C for 3 hours. When the reaction was finished, the methanol solvent was removed using a rotary evaporator (rotation 150 rpm, temperature 40-60°, vacuum 260 mbar) until all the methanol solvent was removed and the remaining solution (around 60 mL distilled water with Pt nanoparticles) was washed with acetone and diethyl ether. Once the washing was finished, the solvent was removed, and the wet solid was dried in an open air with nitrogen flow at room temperature. The black powder was collected and put it in the glass tube and place the glass tube in a vacuum chamber to be dried at room temperature overnight. Before refluxing, the colour of the solution is pale yellow. As the refluxing time increases, the colour of the solution turns into dark brown, suggesting that Pt nanoparticles are formed.[6]

The reaction is following the equation 1 :

$$H_2PtCl_6 + 2CH_3OH \rightarrow Pt^0 + 2HCHO + 6HCl \qquad \textit{equation 1}$$

Monodispersed Pt nanoparticles are difficult to produce. Therefore, a strategy to protect the nanoparticles from agglomeration is important. The linear polymer and micelle are the potential candidates as protective agents to control not only the size but also the shape of the metal nanoparticles.[6]

The structural characterization of the synthetized Pt-NPs prior insertion in the MOFs was performed by using X-ray diffraction. The pattern of the as-synthesized Pt-NPs is displayed in Figure S4.

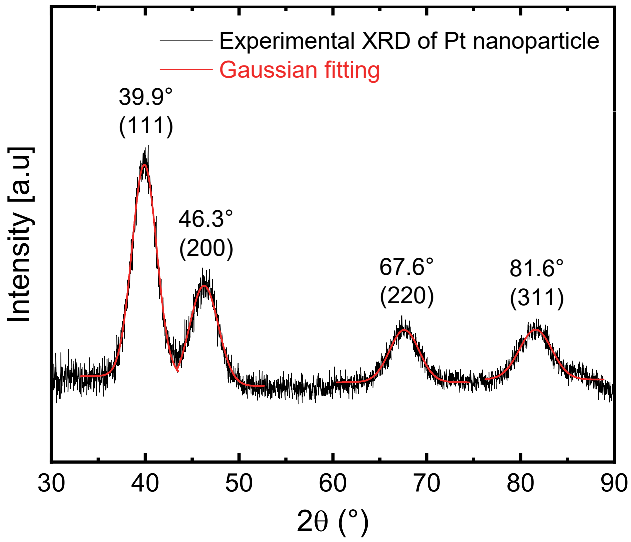

Figure S1. XRD pattern of platinum nanoparticles performed at a wavelength of 0.154 nm. The red line represent the Gaussian fit used to obtain the full-width at half maximum (red).

The broad diffraction peaks confirm the nanosized dimension of the platinum particles. A rough estimation of the platinum crystallite size can be done using the Scherrer formula :

$$D = \frac{K.\lambda}{\beta.cos\theta}$$

where D is the crystallite size, K is a dimensionless factor between 0.8 and 1, λ is the wavelength, β the full-width at half maximum (FWHM), and θ is the diffraction angle. Origin Pro software was used to perform data fitting on the XRD peaks of platinum nanoparticles. A Gaussian fitting function was used and the instrumental broadening was neglected. An average crystallite size around 2.6 nm can be proposed. Details on the calculations for each diffraction line are tabulated in Table S2.

Table S2. Estimation of crystallites size using Scherrer formula (K=0.9)

2theta (°)	39.9	46.3	67.6	81.6
FWHM (°)	3.09714	3.58025	3.7232	4.05993
D (nm)	2.73	2.41	2.57	2.58
average crystallite size = 2.6 nm				

Morphology of platinum nanoparticles was investigated by using a Scanning Transmission Electron Microscope (STEM). Particle size distributions were estimated using ImageJ software. An image of the platinum nanoparticles and the corresponding particle distributions are presented in Fig. S2.

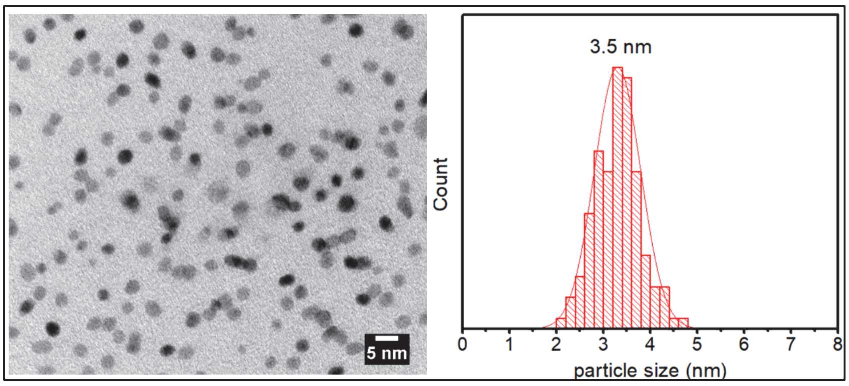

Figure S2. STEM image (left) and particle distributions (right) of Pt-nanoparticles.

As seen in Figure S2, the distribution of Pt-NPs is well described by a normal distribution curve centred at 3.5 nm. This value is larger than the crystallite size obtained from XRD data as mentioned earlier; however, this discrepancy can be linked to several reasons:

- the particles may be polycrystalline
- STEM analysis is performed on a more local selected area of observation, while XRD can be considered as a bulk technique that takes into account average crystallite sizes in the powder samples.[9]
- the instrumental broadening was not considered for the determination of the FWMH which may lead to an under-estimation of the crystallite size.
- the presence of PVP as the capping agent may also increase the size of the observed Pt nanoparticles.

TEM analysis was used to further investigate the structure of the Pt-NPs. The TEM images along with the crystallographic plane analysis are shown in Figure S5. The first insight is that the nanoparticles are mono-crystalline. Distance measurement in STEM images were carried out by using software ImageJ and atomic arrangements models are visualized by using Vesta software.

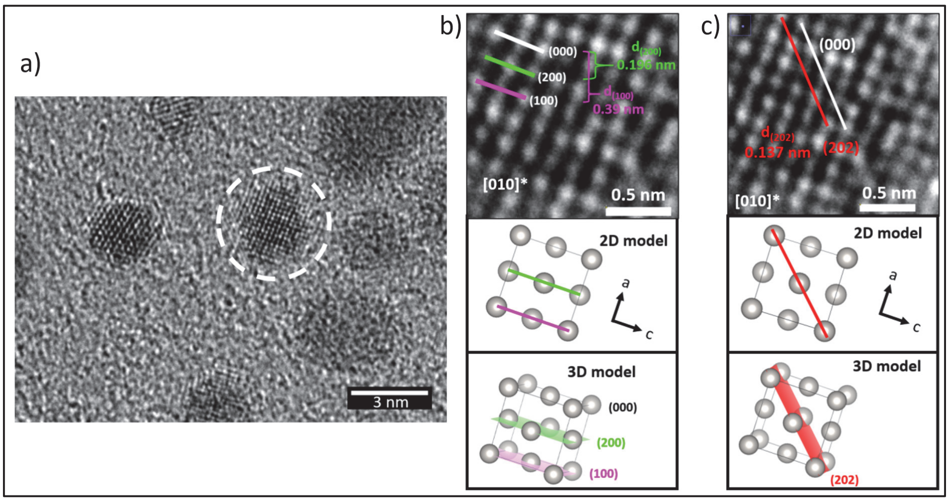

Figure S3. HRTEM images of Pt-NPs showing different crystal plane orientations (a). Pt-NPs viewed from zone axis [010]* to analyze (200) family of plane (b) and (202) family of planes (c) along with their corresponding atomic arrangements models.

TEM analyses revealed that Pt-NPs have various crystal plane orientations. High resolution TEM makes it also possible to measure the interplanar spacing of different planes. The atomic arrangements in the selected area of observation gives useful crystallographic information that complements the XRD analysis. At room temperature, platinum has the face-centered cubic (FCC) crystal system.[10] As shown in Fig. S2, the STEM image shows a crystal plane that coincides with zone axis [010]*. The zone axis (projection vector) and crystal planes are confirmed by measuring the relevant interplanar spacing distances (d) for (200) and (202) family of planes. These family of planes are quite dominants as shown by XRD analysis (Fig. S1). However, the atomic arrangement that corresponds to (111) plane is not observed in this selected particle.

For cubic system, the relationship between interplanar distance (d), lattice parameter (a), and Miller indices (hkl) are written in the following equation.

$$d = \frac{a}{\sqrt{(h^2) + (k^2) + (l^2)}}$$

The lattice parameter of Pt is known to be 0.39 nm.[11] Applying this value in the calculation of interplanar distances for (200) and (220) family of planes results in d values of 0.195 nm and 0.137 nm respectively. These values are in very good agreement with the measured values from the STEM image as depicted in Fig. S6. This confirms the FCC structure of the synthesized Pt-nanoparticles. For better visualization, 2D and 3D models of atomic arrangements in both crystal planes are also displayed in the Fig. S3.

3. Phase purity and crystallinity

Fourier-transform infrared measurements (FTIR) were performed in order to verify the removal of DMF after activation at 373 K. The vibration band corresponding to the C-N vibration of DMF is located at 1256 cm^{-1}. As it can be observed in Fig.S4, this vibration is not observed in any of the synthesized samples. However, FTIR do not allow to conclude about the presence of PVP in the Pt@MOFs, probably because of the encapsulation of Pt(PVP)-NPs inside the MOF crystal.

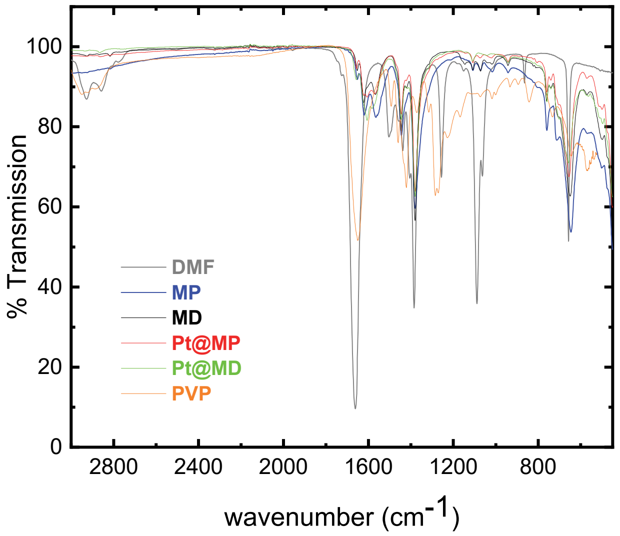

Figure S4. FTIR spectra of DMF and MOF-808 series after activation at 343 K.

X-ray diffraction (XRD) patterns of all the samples are presented in Fig. S5. Only the diffraction peaks corresponding to the cubic Fd-3m structure of MOF-808 are observed as confirmed by the simulated diffractogram [12–14]. It is worth noting that, in the case of Pt@MP and Pt@MD, no Bragg peaks of platinum are evidenced

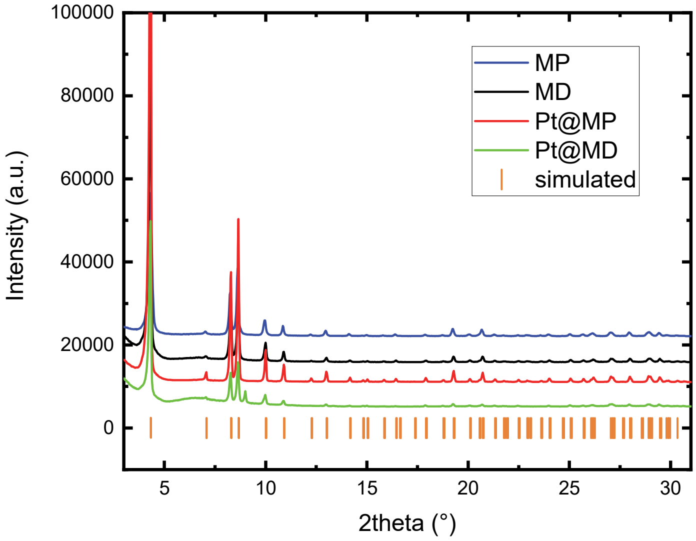

Figure S5. X-ray diffraction pattern measured at 1.54 Å of the samples. The vertical markers refer to the simulated MOF-808 XRD reflections from literature[2] with space group F d-3m and cell parameters a=35.324 Å.

4. Thermal and structural stability

In this section, the methodology used to analyse the MOF decomposition and calculate the number of missing linkers is given.

Theoretical H_2O, OH, and formic acid estimation

For TGA performed under air, in the temperature region between 453°K and 593 K, the degradation could be related to the removal of H_2O, OH or formic acid (FA). Theoretical mass losses for FA, H_2O and OH can be estimated as the following :

Formic acid (FA) = HCOO → M = 45 g/mol
MOF-808 containing coordinated FA = $Zr_6O_4(OH)_4[(C_6H_3)(COO)_3]_2(HCOO)_6$ → M = 1363.7 g/mol
Theoretical FA percent loss → [(6 x 45)/ 1363.7] x 100% = 20%

H_2O → M = 18 g/mol
OH → M = 17 g/mol
MOF-808 containing H_2O and OH that replace FA:
$Zr_6O_4(OH)_4[(C_6H_3)(COO)_3]_2(H_2O)_6(OH)_6$ → M = 1304.09 g/mol
Theoretical H_2O and OH percent loss → [(6 x 18) + (6 x 17) / 1304.9] x 100% = 16%

Theoretical BTC linker estimation

An example of thermogram of pristine MOF-808 (MP) is presented below.

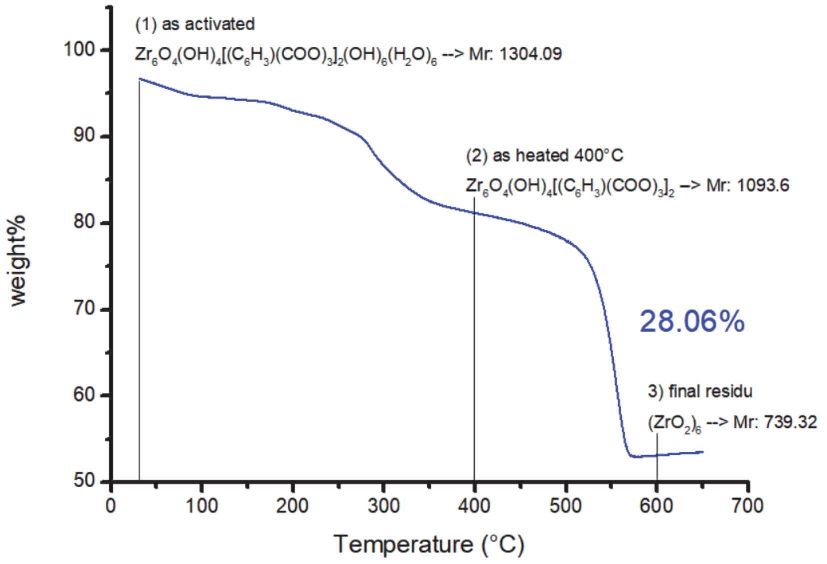

Figure S6. Detailed thermogram of MP.

The as-synthesized sample has the proposed formula as reported in literature[15]: $Zr_6O_4(OH)_4[(C_6H_3)(COO)_3]_2(HCOO)_6$ with M = 1363.7 g/mol. The sample was activated at 100°C under vacuum overnight to remove all solvent and humidity prior to the TGA measurement.

Upon activation, the coordinated formate linkers are removed and upon contact with air, H_2O and OH^- are replacing the position of formate linkers. The proposed activated structure has the formula of $Zr_6O_4(OH)_4[(C_6H_3)(COO)_3]_2(H2O)_6(OH)_6$ with M = 1304.09 g/mol.

For temperature around 400-500°C, coordinated water and hydroxyl are leaving the structure to form $Zr_6O_4(OH)_4[(C_6H_3)(COO)_3]_2$ with M = 1093.6 g/mol. Assuming that the final residual at high temperature is $(ZrO_2)_6$ [16], the theoretical linkers loss can be estimated as the following:

- MOF-808 as-synthesized = $Zr_6O_4(OH)_4[(C_6H_3)(COO)_3]_2(HCOO)_6$, M = 1363.7 g/mol
- MOF-808 activated (100°C) = $Zr_6O_4(OH)_4[(C_6H_3)(COO)_3]_2(H2O)_6(OH)_6$, M = 1304.09 g/mol
- MOF-808 at 400°C = $Zr_6O_4(OH)_4[(C_6H_3)(COO)_3]_2$, M = 1093.6 g/mol

The final structure is $(ZrO_2)_6$ → M = 6 x 123.22 = 739.32

Theoretical BDC loss = $\left(\frac{1093.6 - 739.32}{1093.6}\right) x100\% = 32.4\%$ → correspond to 6 BDC linkers

- BDC loss in pristine MOF-808 (MP) = 28.06% → corresponds to (28.06/32.4) x 6 ≈ 5 BDC linkers, thus 1 missing linker in MP

Applying similar methodology to all MOF-808 samples series, the BDC loss in each sample are presented as the following:

- BDC loss (MD) = 24.74%→ corresponds to (24.74/32.4) x 6 ≈ 4.5 BDC → 1.5 missing linkers
- BDC loss (Pt@MP) = 26.98% → corresponds to (26.98/32.4) x 6 ≈ 5 BDC → 1 missing linkers
- BDC loss (Pt@MD) = 20.43% → corresponds to (20.43/32.4) x 6 ≈ 4 BDC → 2 missing linkers

5. Activity assessment – Water adsorption at 298 K

Water vapor adsorption at 298 K was performed using a BELSORP Max 1. The whole set of adsorption-desorption isotherms is presented in Figure S7/

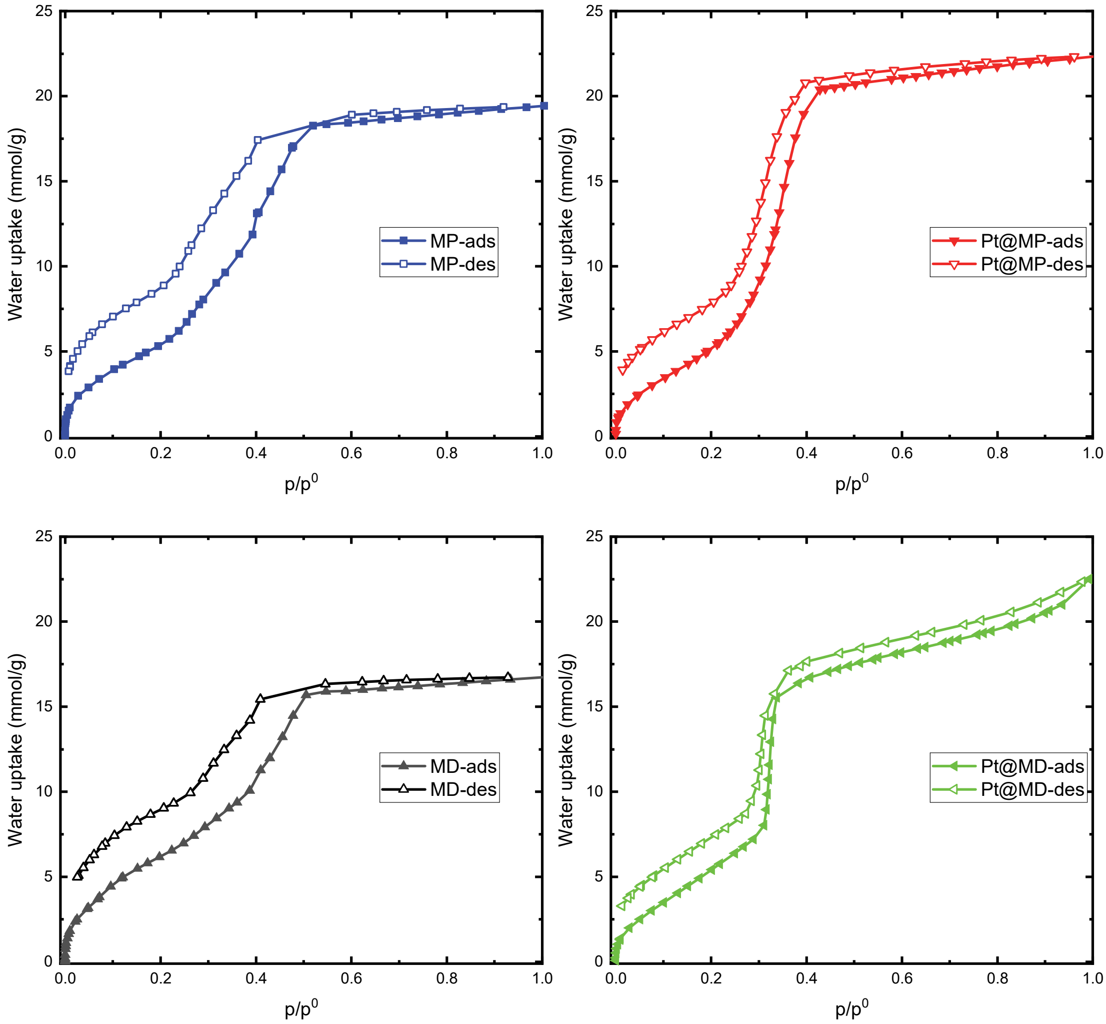

Figure S7. Water adsorption-desorption isotherm measured at 298K for the samples MP, MD, Pt@MP and Pt@MD.

6. References

(1) Furukawa, H.; Gándara, F.; Zhang, Y.-B.; Jiang, J.; Queen, W. L.; Hudson, M. R.; Yaghi, O. M. Water Adsorption in Porous Metal–Organic Frameworks and Related Materials. *J. Am. Chem. Soc.* **2014**, *136* (11), 4369–4381. https://doi.org/10.1021/ja500330a.

(2) Jiang, J.; Gándara, F.; Zhang, Y.-B.; Na, K.; Yaghi, O. M.; Klemperer, W. G. Superacidity in Sulfated Metal–Organic Framework-808. *J. Am. Chem. Soc.* **2014**, *136* (37), 12844–12847. https://doi.org/10.1021/ja507119n.

(3) Healey, K.; Liang, W.; Southon, P. D.; Church, T. L.; D'Alessandro, D. M. Photoresponsive Spiropyran-Functionalised MOF-808: Postsynthetic Incorporation and Light Dependent Gas Adsorption Properties. *J. Mater. Chem. A* **2016**, *4* (28), 10816–10819. https://doi.org/10.1039/C6TA04160D.

(4) Plessers, E.; Fu, G.; Tan, C. Y. X.; De Vos, D. E.; Roeffaers, M. B. J. Zr-Based MOF-808 as Meerwein–Ponndorf–Verley Reduction Catalyst for Challenging Carbonyl Compounds. *Catalysts* **2016**, *6* (7), 104. https://doi.org/10.3390/catal6070104.

(5) Ji, W.; Qi, W.; Tang, S.; Peng, H.; Li, S. Hydrothermal Synthesis of Ultrasmall Pt Nanoparticles as Highly Active Electrocatalysts for Methanol Oxidation. *Nanomaterials* **2015**, *5* (4), 2203–2211. https://doi.org/10.3390/nano5042203.

(6) Teranishi, T.; Hosoe, M.; Tanaka, T.; Miyake, M. Size Control of Monodispersed Pt Nanoparticles and Their 2D Organization by Electrophoretic Deposition. *J. Phys. Chem. B* **1999**, *103* (19), 3818–3827. https://doi.org/10.1021/jp983478m.

(7) Duff, D. G.; Edwards, P. P.; Johnson, B. F. G. Formation of a Polymer-Protected Platinum Sol: A New Understanding of the Parameters Controlling Morphology. *J. Phys. Chem.* **1995**, *99* (43), 15934–15944. https://doi.org/10.1021/j100043a036.

(8) Rösler, C.; Fischer, R. A. Metal–Organic Frameworks as Hosts for Nanoparticles. *CrystEngComm* **2014**, *17* (2), 199–217. https://doi.org/10.1039/C4CE01251H.

(9) Hyde, T. Crystallite Size Analysis of Supported Platinum Catalysts by XRD https://www.technology.matthey.com/article/52/2/129-130/ (accessed Jul 13, 2018).

(10) Smallman, R. E.; Ngan, A. H. W. *Physical Metallurgy and Advanced Materials*; Elsevier: Oxford, 2007.

(11) Arblaster, J. W. Crystallographic Properties of Platinum https://www.technology.matthey.com/article/41/1/12-21/ (accessed Jul 14, 2018).

(12) Plessers, E.; Fu, G.; Tan, C.; De Vos, D.; Roeffaers, M. Zr-Based MOF-808 as Meerwein–Ponndorf–Verley Reduction Catalyst for Challenging Carbonyl Compounds. *Catalysts* **2016**, *6* (7), 104. https://doi.org/10.3390/catal6070104.

(13) Furukawa, H.; Gándara, F.; Zhang, Y.-B.; Jiang, J.; Queen, W. L.; Hudson, M. R.; Yaghi, O. M. Water Adsorption in Porous Metal–Organic Frameworks and Related Materials. *J. Am. Chem. Soc.* **2014**, *136* (11), 4369–4381. https://doi.org/10.1021/ja500330a.

(14) Jiang, J.; Gándara, F.; Zhang, Y.-B.; Na, K.; Yaghi, O. M.; Klemperer, W. G. Superacidity in Sulfated Metal–Organic Framework-808. *J. Am. Chem. Soc.* **2014**, *136* (37), 12844–12847. https://doi.org/10.1021/ja507119n.

(15) Moon, S.-Y.; Liu, Y.; Hupp, J. T.; Farha, O. K. Instantaneous Hydrolysis of Nerve-Agent Simulants with a Six-Connected Zirconium-Based Metal–Organic Framework. *Angewandte Chemie International Edition* **2015**, *54* (23), 6795–6799. https://doi.org/10.1002/anie.201502155.

(16) Valenzano, L.; Civalleri, B.; Chavan, S.; Bordiga, S.; Nilsen, M. H.; Jakobsen, S.; Lillerud, K. P.; Lamberti, C. Disclosing the Complex Structure of UiO-66 Metal Organic Framework: A Synergic Combination of Experiment and Theory. *Chem. Mater.* **2011**, *23* (7), 1700–1718. https://doi.org/10.1021/cm1022882.
